# Socio-Economic Inequalities in Tobacco Consumption of the Older Adults in China: A Decomposition Method

**DOI:** 10.3390/ijerph15071466

**Published:** 2018-07-11

**Authors:** Yafei Si, Zhongliang Zhou, Min Su, Xiao Wang, Dan Li, Dan Wang, Shuyi He, Zihan Hong, Xi Chen

**Affiliations:** 1 School of Public Policy and Administration, Xi’an Jiaotong University, No. 28 Xianning West Road, Xi’an 710049, China; hanshi8860@126.com (Y.S.); sumin1227@126.com (M.S.); danlixjtu@126.com (D.L.); dwang1220@163.com (D.W.); 2 International Business School, Xi’an Jiaotong Liverpool University, Suzhou 215123, China; xiao.wang@xjtlu.edu.cn (X.W.); shuyi.he15@student.xjtlu.edu.cn (S.H.); Zihan.Hong15@student.xjtlu.edu.cn (Z.H.); 3 Department of Health Policy and Management and Department of Economics, Yale University, 60 College Street Suite 301, New Haven, CT 06510, USA; xi.chen@yale.edu

**Keywords:** tobacco consumption, inequality, concentration index, decomposition, China

## Abstract

*Background:* In China, tobacco consumption is a leading risk factor for non-communicable diseases, and understanding the pattern of socio-economic inequalities of tobacco consumption will, thus, help to develop targeted policies of public health control. *Methods:* Data came from the China Health and Retirement Longitudinal Study in 2013, involving 17,663 respondents aged 45 and above. Tobacco use prevalence and tobacco use quantities were defined for further analysis. Using the concentration index (CI) and its decomposition, socio-economic inequalities of tobacco consumption grouped by gender were estimated. *Results:* The concentration index of tobacco use prevalence was 0.044 (men 0.041; women −0.039). The concentration index of tobacco use quantities among smokers was 0.039 (men 0.033; women 0.038). The majority of the inequality could be explained by educational attainment, age, area, and economic quantiles. *Conclusions:* Tobacco consumption was more common among richer compared to poorer people in China. Gender, educational attainments, age, areas, and economic quantiles were strong predictors of tobacco consumption in China. Public health policies need to be targeted towards men in higher economic quantiles with lower educational attainment, and divorced or widowed women, especially in urban areas of China.

## 1. Background

Tobacco consumption is one of the most important risk factor driving the non-communicable diseases (NCDs) epidemic in China [1]. It was estimated that tobacco consumption led to 5.7 million deaths, 6.9% of years of life lost and 5.5% of disability-adjusted life-years (DALYs) worldwide in 2010 [2]. Hence, tobacco control was identified as an immediate priority to reduce NCDs [3]. The widespread socio-economic inequalities in tobacco consumption in developed nations have raised concerns that the overall impact of tobacco is spreading to low- and middle-income countries (LMICs). Therefore, the similar patterns in developed countries may emerge in LMICs [4,5,6], adding economic burdens to the poor populations there.

China is the world’s largest producer and consumer of tobacco, and 44% of the world’s cigarettes are consumed in China. It has been estimated that almost one million lives are lost due to tobacco use per year in China [7,8,9,10]. There is broad consensus that inequalities in health can be partly explained by differences in lifestyles and living conditions [11,12]. Tobacco consumption has been identified as the major cause of inequality in morbidity and mortality. Moreover, tobacco consumption remains remarkably high in groups with low socio-economic status in most high-income countries, contributing to overall inequalities in health [13,14,15]. Therefore, it is likely that the socio-economic inequalities in tobacco consumption are important contributors to the persisting health inequalities in China [12,16].

Smoking cessation may prevent the prevalence of smoking-related diseases even in later mid-life [17], while health effects of tobacco consumption become increasing apparent in middle-aged and older groups [18]. Therefore, advocating cessation in these age groups would be crucial in reducing morbidity and mortality caused by tobacco consumption in the immediate future [19]. Thus, monitoring the socio-economic inequalities in tobacco consumption, and designing distinct anti-smoking policies for specific vulnerable groups, may be a promising approach for equitable distribution of health [14,20,21]. Although extensive literature studied the uneven distribution of tobacco consumption in developed nations, very little has been focused on LMICs [5,6,16,17,18,19,20,21].

This study aims to estimate the distribution of tobacco consumption and the association between tobacco consumption and socio-economic status among older adults (age 45 and above) in China by using a nationally representative cross-sectional data. The objective of this study is to contribute to the literature on the socio-economic inequalities in Chinese tobacco consumption, and to quantify their separate and joint impacts on socio-economic inequalities in older Chinese tobacco consumption.

## 2. Materials and Methods

### 2.1. Data Source

This study used cross-sectional data from the China Health and Retirement Longitudinal Study (CHARLS) conducted in 2013. CHARLS aimed to collect a high-quality nationally-representative sample of Chinese residents aged 45 and older to serve the needs of scientific research. Detailed description of the sampling method, quality assurance measures, and the questionnaire has been previously published [22]. All data will be available for inspection one year after the end of data collection (http://charls.pku.edu.cn/en). After data cleaning (i.e., excluding respondents with illogical answers or with key variables missing), 17,663 respondents were identified for further analysis. Among these, 9213 (52%) were men and 8449 (48%) were women. All analyses of the study were weighted by using individual weights adjusted for non-response to obtain robust results.

### 2.2. Ethics Approval and Consent to Participate

All study procedures were approved by the Health Science Center Ethics Committee at Xi’an Jiaotong University, Shaanxi, China (approval number: 2015-644). Participants provided written consent and parental consent to participate in the study.

### 2.3. Tobacco Consumption Measurement

Three questions in CHARLS were used to capture the information on tobacco consumption of respondents by calculating tobacco use prevalence and tobacco use quantities.

- Question 1: Have you ever chewed tobacco, smoked a pipe, smoked self-rolled cigarettes, or smoked cigarettes/cigars?
- Question 2: Do you still have the habit or have you completely quit?
- Question 3: How many cigarettes do/did you consume approximately per day?

In this study, we defined ‘tobacco use prevalence’ (TP) and ‘tobacco use quantities’ (TQ) to capture tobacco consumption information of the respondents. TQ measured tobacco consumption quantities consumed by smokers, which was deployed to estimate the distribution of tobacco consumption in smoker groups.

### 2.4. Independent Variables

Evidence from economics and economic sociology shows that personal factors, as well as social structural factors may affect the distribution of tobacco consumption [23,24,25]. We included demographic characteristics, socio-economic characteristics, and geographic characteristics of respondents in this study.

Demographic characteristics considered in the study were age group (45–54, 55–64, 65–74, 75 and above), ethnicity (Han ethnicity, Minor ethnicity), marriage status (married, separated, divorced, widowed, or never married et al.), health status (having chronic disease or not), household size and health insurance coverage (UEBMI, URBMI, and NRCMS) [26,27,28,29,30]. The basic health insurance system in China includes the Urban Employee Basic Medical Insurance (UEBMI) since 1998, covering 190 million urban employees, the Urban Resident Basic Medical Insurance (URBMI) since 2007, covering 420 million urban residents, and the New Rural Cooperative Medical Insurance (NRCMI) since 2003, covering 750 million rural residents [30].

Tobacco use is not only closely related to individual demographic characteristics but also socioeconomic factors such as income and educational level. People with lower socioeconomic status are more likely to initiate smoking and less likely to quit smoking than people with higher socioeconomic status in high-income countries [4]. Socio-economic characteristics considered in the study were economic quantiles, educational attainments (illiterate, primary school, middle school, high school and above) and whether the respondent was a communist party member or not [31,32,33]. Economic quantiles were measured by household consumption expenditure per year. Consumption expenditure was used rather than income because income is more likely to be misreported and consumption expenditure is likely a better proxy for resources available [31,32]. We used per-capita consumption expenditure rather than household consumption expenditure to rule out variation in household size while measuring the economic status. We calculated the household consumption expenditure with household living expenditure, such as food, self-produced agricultural products and spending on alcohol in the last week, local transportation, communication, fuels, and entertainment in the last month, as well as education and training, clothing and bedding, and medical expenditure in the last year.

Geographic characteristics consisted of urban-rural distribution and living areas (Northwest—Shaanxi, Gansu, Qinghai, Ningxia and Xinjiang; Southwest—Chongqing, Sichuan, Guizhou, Yunnan and Tibet; South Central—Henan, Hubei, Hunan, Guangdong, Guangxi and Hainan; North—Beijing, Tianjin, Hebei, Shanxi and Inner Mongolia; East—Shanghai, Jiangsu, Zhejiang, Anhui, Fujian, Jiangxi and Shandong; Northeast—Heilongjiang, Jilin and Liaoning).

### 2.5. Statistical Analysis

The inequality of tobacco consumption across socio-economic groups was estimated using a concentration index (CI). It is defined as twice of the area between the concentration curve and the line of equality. CI takes values between −1 and 1, where a positive value indicates that a variable is more concentrated among the richer people, and vice versa [34,35,36,37,38]. The larger the absolute value of CI is, the greater the inequality. The formula for computing the concentration index is:

(1) $$\mathrm{CI}=\frac{2}{\mu }cov\left(y_{i},R_{i}\right)$$

where CI is the concentration index of tobacco consumption, $y_{i}$ is tobacco consumption indicators, *μ* is the mean of tobacco consumption indicators and $R_{i}$ is the fractional rank of household in the economic status distribution. The economic status was measured by the annual household expenditure per-capita.

Inequality can be further explained by decomposing the concentration index into its determining components [35,36,37,38]. Decomposition methods can enable researchers to quantify each determinant’s specific contribution to the measured income-related inequality while controlling for other determinants. The absolute value of contribution signifies the extent to which inequality can be attributed to this variable. A positive contribution to socio-economic inequality means that the relevant variable increases the inequality, and vice versa [35,38]. The linear approximation to the nonlinear model was realized by estimating the partial effects based on the covariate mean values [35,38,39]. As tobacco use prevalence (TP) is a dummy variable, a generalized linear model (GLM) with binomial distribution and identity link was employed to decompose the inequality of tobacco use prevalence [36]. Ordinary least squares (OLS) regression models were employed to decompose the inequality of tobacco use quantities (TQ). The regression model is indicated by Equation (2):

(2) $$y=\alpha^{m}+\sum_{j}\beta_{j}^{m}x_{j}+\varepsilon$$

where *y* is tobacco consumption indicator, $\beta_{m}^{j}$ is partial effects (i.e., d*y*/d*x_j_*) of each variable and evaluated at sample means; $\alpha^{m}$ is the constant term in the regression equation, ε is the error term. Calculating the concentration index of Equation (2) and the decomposition of the concentration index CI could be specified as:

(3) CI=∑j(βjmxj¯μ)Cj+GCε

where *μ* is the mean of the dependent variable, *C_j_* is the concentration index for *x_j_*, $\bar{x_{j}}$ is the means of *x_j_* and βjmxj¯μ is the elasticity of $x_{j}$ in tobacco consumption and *G* is the elasticity of ε in tobacco consumption. The contribution of $x_{j}$ is defined as the product of the elasticity of $x_{j}$ in tobacco consumption and the concentration index of $x_{j}$. The first term on the right side of Equation (3) denotes the contribution of observable variables to inequality in tobacco consumption and the last term is the contribution of ε, which cannot be directly calculated. The percentage of contribution of $x_{j}$ is defined as the contribution of $x_{j}$ divided by CI. Theoretically, the contribution of $x_{j}$ is likely to exceed one hundred percent because only income-related inequalities were measured by CI. The total contribution to CI of $x_{j}$ and the error term ε is definitely one hundred percent. Moreover, the contribution of each determinant to the change of the concentration index of tobacco consumption can be attributed to an interaction of changes, including the change of this determinant, the change of the determinant’ concentration index, and the change of partial effects of the determinant on tobacco consumption [38]. All analyses were performed with Stata 13.0 (Stata Corp. LP, College Station, TX, USA).

## 3. Results

Table 1 shows summary statistics for tobacco use prevalence, number of cigarettes per day among smokers, and independent variables. Roughly 16.0% of respondents were reported to consume tobacco in China now (men: 29.7%; women: 3.5%). As for quantities of tobacco consumption, the smokers on average consumed 14.4 cigarettes per day (men: 14.9; women: 10.7). A total of 64.6% of the respondents had chronic diseases (men: 62.8%; women: 66.2%) and 25.3% of the respondents never attended school (men: 11.9%; women: 37.7%). Moreover, 11.7% of the respondents did not have a spouse (men: 8.4%; women: 14.8%), including either separated, divorced, widowed, or never married at all.

Table 2 shows tobacco consumption among the older adults (aged 45 and above) in different economic quantiles in China. Among men, the richest had the highest probability of being smokers (32.7%), while the poorest had the lowest probability (26.0%). However, the poorest women had the highest tobacco use prevalence (4.1%) while lower among the poorer women (3.0%). Among men, statistically significant differences in tobacco use prevalence were observed in different economic quantiles. Among women, no statistical significance in tobacco use prevalence was observed across different economic quantiles. The pattern was similar to tobacco use quantities.

The inequalities of tobacco consumption for the older adults (aged 45 and above) in China were measured by the concentration index (Table 3). The CI of tobacco use prevalence (TP) was 0.044 (95% confidence interval: 0.024–0.064) for all older adult and 0.041 (95% confidence interval: 0.022–0.060) for men. The CIs were statistically significant, indicating that the richer had a higher prevalence of smoking compared to the poor. Among women, the CI of tobacco use prevalence was not statistically significant, indicating there was no difference in tobacco use prevalence in different economic quantiles. As for tobacco use quantities in smokers (TQ), the significantly positive value of the concentration indices (0.055 and 0.098) indicated strong pro-rich inequalities in tobacco consumption among older adults in China.

Inequalities of tobacco consumption can be further explained by decomposing the concentration indices into their determined components. We present the partial effect (d*y*/d*x*), the contribution to concentration index (Cont.) and the percentage of contribution (%) of each determined component in Table 4 and Table 5.

Compared with the poorest quantile, the prevalence of tobacco use in the richest quantile was 3.3% higher in the whole population and was 5.7% higher among men. Meanwhile, the richer smoked more than the poorer among smokers (the poorer to the richest: 2.1, 2.7, 3.3, and 4.8). Furthermore, those who attended primary school (−1.9%), middle school (−3.0%), and high school and above (−5.0%) had a lower probability of tobacco use than those who had never attended school. However, when examining cigarette consumption in the whole population, the results suggested that those who attended primary school consumed more among the smokers (cigarettes: 1.9) compared to those who never attended school. Taking the age group of 45–54 years as a reference, the probability of tobacco consumption was lower in the age group 55–64 (−4.7%), 65–74 (−6.4%), and 75 and above (−10.2%). Among smokers, members of the age group 65–74 (cigarettes: −1.3) and 75 and above (cigarettes: −4.5) consumed less than the age group 45–54. With regard to marital status, smoking prevalence and the number of cigarettes consumed per day among those who were separated, divorced, widowed, or never married were higher than the ones among those who were married. After decomposing the concentration indices (Table 4 and Table 5), the income-related inequalities were decomposed into the contributions of different variables. The majority of inequality in tobacco use prevalence (TP) can be attributed to the richest quantile (104.5%), being men (−61.1%), having high school education or above (−45.6%), or being 66–74 or above 75 years of age (20.6% and 23.4%, respectively). The total contribution of observables is −4.82%, which means that 104.82% of the positive contribution to inequality in incidence is explained in the error term of the regression. The majority of inequality in tobacco use quantities among the smokers (TQ) can be attributed to being in the richest quantile (93.8%), being in the richer quantile (30.6%), being in the poorer quantile (−20.2%), being 75 years of age or above (11.0%), or being married (−6.7%). The total contribution of observables is 101.46%, which means that 1.46% of the negative contribution to inequality is explained by the error term of the regression.

## 4. Discussion

Involving more than 17,600 men and women respondents aged 45 and above in China, our study finds that high-income residents had a higher tobacco use prevalence, and consumed more tobacco among smokers, especially for men. For tobacco use prevalence and tobacco use quantities among smokers, the results also indicated a significant gap between men and women in China. Our results also identified several key socio-economic variables associated with tobacco consumption among the older adults (aged 45 and above) in China. As highlighted by other researchers, gender, age, and economic status are the key indicators for monitoring the inequalities in tobacco consumption [17,18,19,20,40]. Public health policies need to be targeted towards the least educated and the oldest groups.

The cross-national tobacco use often served as the starting point to explain socio-economic inequalities in tobacco consumption [4,17,19,26]. The price effect is strong in low-income nations, implying that cigarettes become more affordable as personal and family incomes rise, while the health-cost effect rises when income grows, suggesting that tobacco consumption declines as personal and family incomes rise in high-income countries, where smoking’s greater cost becomes more salient. Consequently, pro-rich inequalities in tobacco consumption in low-income countries and pro-poor inequalities in tobacco consumption in high-income countries were observed in prior studies, while inequalities in tobacco consumption in middle-income countries varied greatly [4,5,13,17,19]. Our results indicated that the large majority of the low-income residents had a lower probability of tobacco use and consumed less tobacco even if they smoked, owing to the lack of resources. As household per-capita income grows, however, residents who are used to being price-sensitive can better afford manufactured cigarettes, as well as their stimulating and addictive properties. Thus, residents in higher economic quantiles consumed more tobacco among older adults in China.

Another important finding in our study was the different and even contradictory effects of household economic quantiles and individual educational attainment on tobacco consumption among older adults [27]. Those with a higher economic quantile, but are less educated, consumed more tobacco than those with a lower economic quantile or with higher educational attainment. One plausible explanation for the difference is that the price effect is still strong in China, especially among older adults since most of the elderly would face a sharp decline of income after retirement. Thus, tobacco for those in a lower economic quantile with limited resources becomes less affordable. Meanwhile, our study also revealed that the prevalence of tobacco consumption was generally higher among less educated groups who have less information or knowledge about the effects of smoking. A majority of the residents, even the ones with none or limited formal education, benefited from the fast growth of the Chinese economy and grasped the opportunity to achieve a higher economic quantile during the past forty years [41]. However, their health status did not improve with the changing of their economic quantiles due to the lack of health-related information and knowledge, such as addiction to tobacco consumption [42].

The results also indicated a significant gap of tobacco consumption between men and women in China, and both the pattern and magnitude of inequality varied greatly [13,18,40]. Men had significantly higher tobacco use prevalence and consumed more tobacco than women. The positive association between economic quantiles and tobacco consumption were observed among male smokers, while only positive association of tobacco use quantities and economic quantiles was found among female smokers. Tobacco consumption of women may also derive from factors beyond economic status. Our results indicated that the marriage status (e.g., being separated, divorced, widowed, or never married) was significantly associated with women using tobacco, with a lower probability, but heavier tobacco consumption, compared to men, especially in urban areas of China. Moreover, the effect of education on tobacco consumption diminished among women compared to men. Hence, it is rather necessary to develop gender-specific policies to efficiently reduce the inequalities in tobacco consumption in China. The different geographic concentration pattern of tobacco consumption among men and women implied that the control of tobacco consumption among men should be strengthened in south-central and northern regions, while control policies for women’ consumption should be strengthened in eastern and northeastern regions.

Given that China announced a new tobacco tax structure and intended to increase retail prices by 3.4% in 2009 [43], and that the self-reported tobacco consumption may also lead to a lower tobacco use prevalence, our results indicated a lower tobacco use prevalence (16.0%) among elderly in China (29.7% of men and 3.5% of women) and an average consumption of 14.2 cigarettes for smokers per day (14.3 among men and 10.6 among women) [7,8]. The Sustainable Development Goals (SDGs) now request China to reduce premature deaths attributed to NCDs by 33.3% by 2030 [10]. Despite tobacco consumption being a leading risk factor for the major NCDs, the burden of NCDs in China is enormous and the epidemic of tobacco consumption is at the highest level in the world [3]. Largely depending on the social call to control tobacco consumption [10], China remains the largest consumer of tobacco worldwide and a tobacco tax is still at a rather low level [7,8,9,10]. It is apparent that the competing priorities between interests of the tobacco industry and protecting the population’s health are entangled in China [9,10,44].

Raising tobacco taxes has been deemed as one of the most cost-effective measures to reduce tobacco consumption, and to generate substantial revenue for health and other infrastructure programs that ultimately benefit the entire population [45,46,47]. Two rationales against raising tobacco tax—the unfair burden on low-income smokers, and consumers’ ability of switching to cheaper tobaccos [22]—however, have always been referred with the assumption that the poorest groups consumed more tobacco than the rich did [6,48,49]. Notwithstanding, our findings highlighted that the increase of economic quantiles was positively associated with higher tobacco use prevalence and more tobacco consumption. Therefore, the pro-rich distribution of tobacco use prevalence (TP) and tobacco use quantities in smokers (TQ) questioned the rationale against raising the tobacco tax, while supporting the rationale of raising tobacco tax among the older population in China. Given the well-document studies suggesting the health effects of tobacco cessation among adults, targeted cessation in this group would be extremely important, as a component of overall policy initiatives for reducing tobacco consumption epidemic in the nation [19]. This will be crucial for reducing morbidity and mortality caused by tobacco consumption in the near future.

Our study also highlighted some important strengths. To the best of our knowledge, this is the first study to explore income-related inequalities in tobacco consumption in China. Given tobacco consumption is a health risk, monitoring the distribution and intensity of tobacco consumption is critical for identifying potential issues for developing policies [20]. China has the largest number of smokers in the world. Reducing tobacco consumption will significantly decrease the global burden of tobacco-related illnesses and deaths [44]. Using large and representative samples of the Chinese older population (aged 45 and above) in 2013, and following the global pattern of tobacco consumption by gender, this paper added to the literature by measuring gender-specific concentration indices for two smoking-related variables from CHARLS (TP and TQ). Our study, thus, provided a more detailed picture of the inequalities in Chinese tobacco consumption. Finally, studies of inequalities in tobacco consumption in China will be of great importance to other developing countries since China shares many societal and economic challenges they also face.

Some limitations in this study should be noticed as well. Firstly, the use of self-reported measures may substantially underestimate or overestimate tobacco use prevalence (TP) and tobacco use quantities (TQ) in tobacco consumption. For instance, the low prevalence among the oldest age group may be due to higher death rates of smokers among the elderly, leading to the underestimation of tobacco consumption among the oldest age group. Secondly, self-reported consumption expenditure may also be underestimated in this study. Ideally it might be better to use a per-capita consumption variable using adult equivalent scales rather than number of household members. However, numbers of children in household cannot be identified because of the survey design [50,51]. Thirdly, owing to data availability, not all factors were included in this study, such as occupation of the respondents and some potential unobservable respondents’ characteristics. Omission of these factors could lead to biased estimation of the inequality in tobacco consumption. Finally, our results also supported the idea that there are multiple, and sometimes even opposite forces that cause social inequalities in tobacco consumption [26]. Income or consumption often serve as a proxy for socio-economic position, while the concentration index of tobacco consumption based on income or consumption is limited for measuring all socio-economic inequalities in tobacco consumption.

## 5. Conclusions

Our results indicated that there were significantly pro-rich inequalities of tobacco consumption in older adults in China, implying that richer people consumed more tobacco than poorer. Furthermore, gender, education attainment, age, area, and economic quantiles were strong predictors of tobacco consumption. Both the pattern and magnitude of inequality in tobacco consumption between men and women varied significantly. Hence, it is quite necessary to develop gender-specific policies to efficiently reduce tobacco consumption. Public health policies need to be targeted towards men of higher economic status with lower educational attainment, as well as divorced or widowed women, especially in urban areas of China. The different geographic concentration pattern of tobacco consumption among men and women implied that the control of tobacco consumption among men should be strengthened in south-central and northern regions, while control policies for females’ consumption should be strengthened in eastern and northeastern regions.

## Figures and Tables

**Table 1:** Variables description and descriptive analysis.

Variables	Variables Description	Mean/%		
		Men	Women	All
Tobacco use prevalence (TP)	Tobacco use or not	29.7	3.5	16.0
Tobacco use quantities (TQ)	Number of cigarettes per day among smokers	14.9	10.7	14.4
Chronic				
No ^†^	Don’t have chronic diseases = 1, no = 0	37.2	33.8	35.5
Yes	Having chronic diseases = 1, no = 0	62.8	66.2	64.5
Age group				
45–54 ^†^	45 ≤ Age ≤ 54	30.7	35.2	33.0
55–64	55 ≤ Age ≤ 64	38.0	37.0	37.5
65–74	65 ≤ Age ≤ 74	22.0	19.8	20.7
75 and above	75 ≤ Age	9.3	8.3	8.8
Party				
No ^†^	Communist = 0, no = 1	82.3	95.7	89.3
Yes	Communist = 1, no = 0	17.7	4.3	10.7
Urban				
Urban	Urban = 1, else = 0	39.3	40.4	39.9
Rural ^†^	Rural = 1, else = 0	60.7	59.6	60.1
Educational attainment				
Illiteracy ^†^	No formal education(illiterate) = 1, else = 0	11.9	37.7	25.3
Primary school	Primary school = 1, else = 0	44.1	36.7	40.2
Middle school	Middle school = 1, else = 0	26.8	16.4	21.4
High school and above	High school and above = 1, else = 0	17.2	9.2	13.0
Household size	Amount of household members	4.4	4.4	4.4
Ethnicity				
Minor ethnicity ^†^	Minor nationality = 1, else = 0	6.9	8.3	7.7
Han ethnicity	Han nationality = 1, else = 0	93.1	91.7	92.3
Marriage				
Married ^†^	Married = 1, Divorce or else = 0	91.6	85.2	88.3
Divorce or else	Divorce or else = 1, married = 0	8.4	14.8	11.7
Basic insurance				
UEBMI ^†^	Having UEBMI = 1, no = 0	16.1	10.9	13.4
URBMI	Having URBMI = 1, no = 0	6.8	8.7	7.8
NRCMS	Having NRCMS = 1, no = 0	77.1	80.4	78.8
Area				
Northwest ^†^	Living in northwest of China = 1, else = 0	7.1	7.2	7.2
Southwest	Living in southwest of China = 1, else = 0	17.5	17.4	17.4
South middle	Living in south middle of China = 1, else = 0	23.9	24.1	24.0
North	Living in north of China = 1, else = 0	13.5	13.2	13.4
East	Living in east of China = 1, else = 0	7.6	7.7	7.6
Northeast	Living in northeast of China = 1, else = 0	30.4	30.4	30.4

Notes: ^†^ Reference levels in the regressions; UEBMI: the Urban Employment Basic Medical Insurance; URBMI: the Urban Resident Basic Health Insurance; NRCMS: the New Rural Cooperative Medical Scheme. Mean was calculated for continuous variables and percentage (%) was used for category variables.

**Table 2:** Tobacco use prevalence and tobacco use quantities for the older adults in different economic quantiles in China.

Economic Quantiles	Tobacco Use Prevalence (TP)			Tobacco Use Quantities (TQ)		
	Men	Women	All	Men	Women	All
Poorest	26.0	4.1	14.0	12.6	8.4	11.9
Poorer	29.0	3.0	15.5	14.2	12.3	14.0
Middle	30.9	4.0	17.0	14.8	11.1	14.4
Richer	28.8	3.0	15.6	15.8	10.1	15.2
Richest	32.7	3.4	17.7	16.5	11.8	16.0
*p*-value	<0.001	0.179	<0.001	<0.001	0.081	<0.001

Notes: Univariate ANOVAS was employed for continuous variables (TQ) and chi-squared test was used for the dummy variable (TP).

**Table 3:** Concentration index of smoking incidence and tobacco use quantities for the older adults in China.

Groups	Tobacco Use Prevalence (TP)			Tobacco Use Quantities (TQ)		
	CI	95% Confidence Interval		CI	95% Confidence Interval	
		Lower Limit	Higher Limit		Lower Limit	Higher Limit
Men	0.041	0.022	0.060	0.051	0.033	0.069
Women	−0.039	−0.102	0.023	0.056	0.003	0.110
All	0.044	0.024	0.064	0.055	0.038	0.072

**Table 4:** Decomposition of concentration index of tobacco use prevalence (TP).

Independent Variables	Men			Women			All		
	d*y*/d*x*	Cont.	%	d*y*/d*x*	Cont.	%	d*y*/d*x*	Cont.	%
Economic quantiles (Poorest ^†^)									
Poorer	0.0175	−0.0039	−9.27	−0.0064	0.0119	−29.79	0.0026	−0.0010	−2.40
Middle	0.0293 *	−0.0070	−16.83	0.0051	−0.0102	25.65	0.0150 *	−0.0067	−15.27
Richer	0.0168	−0.0016	−3.71	−0.0062	0.0048	−12.08	0.0040	−0.0007	−1.58
Richest	0.0568 ***	0.0431	103.20	0.0078	0.0494	−124.02	0.0325 ***	0.0456	104.53
Chronic	−0.0112	0.0000	0.00	0.0037	0.0000	0.00	−0.0024	0.0000	0.00
Education (Illiteracy ^†^)									
Primary school	−0.0160	0.0021	4.98	−0.0125 **	0.0136	−34.16	−0.0190 **	0.0046	10.49
Middle school	−0.0413 **	0.0017	3.96	−0.0141 **	0.0047	−11.86	−0.0297 ***	0.0022	5.05
High school and above	−0.0663 ***	−0.0144	−34.45	−0.0259 ***	−0.0471	118.36	−0.0495 ***	−0.0199	−45.57
Age (45–54 ^†^)									
55–64	−0.1086 ***	−0.0053	−12.72	0.0055	0.0023	−5.66	−0.0470 ***	−0.0043	−9.76
65–74	−0.1427 ***	0.0108	25.87	0.0080	−0.0051	12.71	−0.0641 ***	0.0090	20.56
75 and above	−0.2025 ***	0.0110	26.22	−0.0010	0.0004	−1.11	−0.1022 ***	0.0102	23.44
Housize	0.0084 **	−0.0049	−11.82	0.0005	−0.0025	6.23	0.0042 **	−0.0045	−10.41
Male							0.2684 ***	−0.0267	−61.08
Party	−0.0115	−0.0009	−2.18	−0.0068	−0.0045	11.35	−0.0187 *	−0.0028	−6.31
Area (Northwest ^†^)									
Southwest	0.0001	0.0000	0.00	−0.0051	0.0009	−2.16	−0.0026	0.0001	0.22
South middle	−0.0616 ***	0.0054	12.91	0.0006	−0.0005	1.19	−0.0282 **	0.0046	10.48
North	−0.0980 ***	−0.0046	−11.06	0.0511 ***	0.0202	−50.72	−0.0174	−0.0015	−3.48
East	−0.0521 *	−0.0002	−0.37	0.0432 ***	0.0011	−2.70	−0.0002	0.0000	0.00
Northeast	−0.0899 ***	−0.0076	−18.30	0.0088	0.0063	−15.81	−0.0380 ***	−0.0060	−13.70
Insurance (UEBMI ^†^)									
URBMI	−0.0200	0.0000	0.00	0.0170 *	0.0000	0.00	0.0083	0.0000	0.00
NRCMS	0.0281	0.0000	0.00	0.0295 ***	0.0000	0.00	0.0333	0.0000	0.00
Urban	−0.0066	−0.0008	−1.87	0.0138 ***	0.0137	−34.46	0.0067	0.0015	3.34
Marriage	0.0372 *	−0.0019	−4.58	0.0143 **	−0.0062	15.45	0.0381 ***	−0.0036	−8.33
Ethnicity	0.0696 ***	−0.0023	−5.58	0.0086	−0.0024	6.08	0.0355 ***	−0.0022	−5.04
Total contribution		0.0185	44.4		0.0508	−127.48		−0.0021	−4.82
Contribution of ε		0.0232	55.6		−0.0906	227.48		0.0457	104.82

Notes: * *p* < 0.1; ** *p* < 0.05; *** *p* < 0.01; d*y*/d*x* means partial effects of each variable and evaluated at sample means; Cont. means contribution to CI; % means the percentage of contribution to CI; ^†^ Reference levels in the regressions; UEBMI: the Urban Employment Basic Medical Insurance; URBMI: the Urban Resident Basic Health Insurance; NRCMS: the New Rural Cooperative Medical Scheme.

**Table 5:** Decomposition of concentration index of tobacco use quantities in smokers (TQ).

Independent Variables	Men			Women			All		
	d*y*/d*x*	Cont.	%	d*y*/d*x*	Cont.	%	d*y*/d*x*	Cont.	%
Economic quantiles (Poorest ^†^)									
Poorer	1.9330 **	−0.0098	−19.13	3.3471 *	−0.0237	−42.20	2.1137 ***	−0.0111	−20.24
Middle	2.6193 ***	−0.0007	−1.28	3.4425	−0.0012	−2.14	2.7401 ***	−0.0007	−1.29
Richer	3.5085 ***	0.0172	33.50	1.1368 **	0.0078	13.83	3.3155 ***	0.0168	30.63
Richest	4.8506 ***	0.0501	97.69	4.3576 **	0.0627	111.78	4.8122 ***	0.0513	93.77
Chronic	0.3361	0.0002	0.35	−0.6612	−0.0005	−0.88	0.2733	0.0002	0.28
Education (Illiteracy ^†^)									
Primary school	2.0859 **	−0.0023	−4.50	2.0613	−0.0032	−5.67	1.8581 **	−0.0021	−3.88
Middle school	0.7611	0.0012	2.28	−0.3816	−0.0008	−1.46	0.4499	0.0007	1.30
High school and above	−0.2938	−0.0008	−1.48	2.3132	0.0083	14.85	−0.5354	−0.0014	−2.61
Age (45–54 ^†^)									
55–64	0.4546	0.0000	0.06	2.0961	0.0002	0.35	0.6106	0.0000	0.08
65–74	−1.2290	0.0015	3.01	−0.5932	0.0010	1.85	−1.2775 *	0.0017	3.02
75 and above	−4.8545 ***	0.0063	12.21	−1.8228	0.0033	5.84	−4.5358 ***	0.0060	11.04
Housize	0.1175	−0.0008	−1.54	0.7644 **	−0.0072	−12.79	0.1849	−0.0013	−2.35
Male							4.5376 ***	0.0023	4.17
Party	1.2449 *	0.0017	3.35	−3.0785	−0.0059	−10.55	1.1095	0.0016	2.89
Area (Northwest ^†^)									
Southwest	−0.8954	0.0002	0.45	−0.7127	0.0003	0.45	–0.9002	0.0002	0.43
South middle	−0.4308	0.0002	0.31	0.5983	−0.0003	−0.55	−0.3973	0.0002	0.28
North	1.3851	0.0008	1.63	5.2067 *	0.0044	7.81	1.4707	0.0009	1.68
East	−0.7965	−0.0004	−0.78	6.0109 **	0.0042	7.54	0.0456	0.0000	0.04
Northeast	2.3621 **	−0.0006	−1.22	5.8943 **	−0.0022	−3.88	2.5736 ***	−0.0007	−1.29
Insurance (UEBMI ^†^)									
URBMI	1.7689	0.0011	2.13	−2.7413	−0.0024	−4.21	1.1634	0.0007	1.36
NRCMS	0.7765	−0.0030	−5.93	1.5085	−0.0082	−14.68	0.7239	−0.0029	−5.35
Urban	−0.5256	−0.0020	−3.99	−1.3910	−0.0075	−13.45	−0.6062	−0.0024	−4.45
Marriage	2.0798 **	−0.0030	−5.78	4.3339 ***	−0.0086	−15.35	2.4717 ***	−0.0036	−6.65
Ethnicity	2.2198 **	−0.0008	−1.48	0.9094	−0.0004	−0.77	2.1428 **	−0.0008	−1.38
Total contribution		0.0563	109.84		0.0200	35.72		0.0555	101.46
Contribution of ε		−0.0050	−9.84		0.0360	64.28		−0.0008	−1.46

Notes: * *p* < 0.1; ** *p* < 0.05; *** *p* < 0.01; d*y*/d*x* means partial effects of each variable and evaluated at sample means; Cont. means contribution to CI; % means the percentage of contribution to CI; ^†^ Reference levels in the regressions; UEBMI: the Urban Employment Basic Medical Insurance; URBMI: the Urban Resident Basic Health Insurance; NRCMS: the New Rural Cooperative Medical Scheme.
